# Neuronal-activity regulated gene expression: emphasis on BDNF

**DOI:** 10.1186/2193-1801-4-S1-L38

**Published:** 2015-06-12

**Authors:** Tõnis Timmusk

**Affiliations:** Tallinn University of Technology, Estonia

**Keywords:** BDNF, synaptic plasticity, gene regulation

Neurotrophin brain-derived neurotrophic factor (BDNF) is a growth factor that has important roles in the development and functioning of nervous system by promoting the survival, differentiation and synaptic plasticity of specific neuronal populations. Modifiability of neuronal connectivity by formation of new synapses, and alteration of the strength and stability of existing synapses, is regarded as the main cellular basis for memory and long-term behavioral adaptations. The gene encoding BDNF is considered to be one of the master genes of synaptic plasticity. BDNF has also received particular interest for its deregulation in nervous system disorders. Decreases of BDNF and its receptor TrkB levels and activity are accompanied by and are believed to lead to several pathologies, particularly nervous system diseases like neurodegenerative, psychiatric and cognitive diseases. Therefore knowledge about the regulatory mechanisms of BDNF gene is important both for understanding of nervous system function and for finding new drug targets. Results of our studies on the molecular mechanisms of neuronal activity-regulated expression of BDNF gene in the nervous system will be presented and discussed.

